# Load-Relaxation Properties of the Human Trunk in Response to Prolonged Flexion: Measuring and Modeling the Effect of Flexion Angle

**DOI:** 10.1371/journal.pone.0048625

**Published:** 2012-11-05

**Authors:** Nima Toosizadeh, Maury A. Nussbaum, Babak Bazrgari, Michael L. Madigan

**Affiliations:** 1 Department of Industrial and System Engineering, Virginia Tech, Blacksburg, Virginia, United States of America; 2 Department of Biomedical Engineering and Sciences, Virginia Tech, Blacksburg, Virginia, United States of America; 3 Department of Biomedical Engineering, University of Kentucky, Lexington, Kentucky, United States of America; 4 Department of Engineering Science and Mechanics, Virginia Tech, Blacksburg, Virginia, United States of America; Semmelweis University, Hungary

## Abstract

Experimental studies suggest that prolonged trunk flexion reduces passive support of the spine. To understand alterations of the synergy between active and passive tissues following such loadings, several studies have assessed the time-dependent behavior of passive tissues including those within spinal motion segments and muscles. Yet, there remain limitations regarding load-relaxation of the lumbar spine in response to flexion exposures and the influence of different flexion angles. Ten healthy participants were exposed for 16 min to each of five magnitudes of lumbar flexion specified relative to individual flexion-relaxation angles (i.e., 30, 40, 60, 80, and 100%), during which lumbar flexion angle and trunk moment were recorded. Outcome measures were initial trunk moment, moment drop, parameters of four viscoelastic models (i.e., Standard Linear Solid model, the Prony Series, Schapery's Theory, and the Modified Superposition Method), and changes in neutral zone and viscoelastic state following exposure. There were significant effects of flexion angle on initial moment, moment drop, changes in normalized neutral zone, and some parameters of the Standard Linear Solid model. Initial moment, moment drop, and changes in normalized neutral zone increased exponentially with flexion angle. Kelvin-solid models produced better predictions of temporal behaviors. Observed responses to trunk flexion suggest nonlinearity in viscoelastic properties, and which likely reflected viscoelastic behaviors of spinal (lumbar) motion segments. Flexion-induced changes in viscous properties and neutral zone imply an increase in internal loads and perhaps increased risk of low back disorders. Kelvin-solid models, especially the Prony Series model appeared to be more effective at modeling load-relaxation of the trunk.

## Introduction

Trunk flexion exposures, whether prolonged or cyclic, result in viscoelastic deformation of passive tissues in the posterior trunk and consequently a reduction in trunk stiffness [1], [2]. A decrease in passive trunk stiffness can be compensated by extra activation of muscles [3]–[6], which may cause additional loads on joints and other soft tissues [7]. Moreover, extra activation of muscles may increase metabolic cost and consequently contribute to muscle fatigue [4], [8]. Since the risk of low back disorders (LBDs) may be associated with excessive spinal loads and muscle fatigue [9]–[11], an accurate assessment of the time-dependent changes in load partitioning among passive trunk tissues and active muscles is of importance in investigating the risk of LBDs.

Determining the distribution of loads among passive and active components of the human trunk, typically using a biomechanical model, requires a realistic representation of time-dependent passive properties. A number of experiments have assessed the time-dependent behavior of passive trunk tissues. Many *in vitro* studies have focused on the viscoelastic properties of spinal motion segments, especially in flexion/extension [12]–[16]. Several other studies have determined the viscoelastic properties of muscle using both *in vitro* [17]–[23] and *in vivo* [24]–[30] measurements. Furthermore, in an *in vivo* study by McGill and Brown [31], the whole-trunk creep was measured for prolonged flexion exposures.

While these studies have provided a fundamental understanding of the time-dependent responses of trunk tissues, some limitations still exist. Most measurements of the viscoelastic properties of the spine have been performed on cadaver motion segments. The main limitation of these *in vitro* experiments is the lack of metabolic processes of intervertebral discs, respiration, circulation and muscle activity, which are influential in prolonged tests [32], [33]. Many occupational tasks require prolonged trunk flexion at a constant angle (load-relaxation); however, no studies to our knowledge have measured load-relaxation of the lumbar spine *in vivo* in response to flexion exposures. Previous reports show that load-relaxation behavior of soft tissues is not directly correlated to creep response [34], [35], which indicates that load-relaxation is not simply the inverse of creep responses and that they should be determined separately. Furthermore, there is evidence of nonlinear viscoelastic behaviors for spinal soft tissues and motion segments [33], [36], [37]. However, it is unknown how such nonlinearity in viscoelastic behavior is influenced by different magnitudes of loading/displacement.

Hence, the main purpose of this study was to quantify the load-relaxation responses of the human trunk during prolonged flexed postures. Load-relaxation responses were measured *in vivo* at several lumbar flexion angles and then fit using a range of viscoelastic models. Based on previous evidence of nonlinear viscoelastic behavior of trunk soft tissues [33], [36], [37], we hypothesized that the whole trunk would exhibit nonlinear viscoelastic responses to prolonged flexion and that these responses would depend on the specific lumbar flexion angle. Several different approaches, based on equations of creep deformation or load-relaxation, have been previously developed to model the viscoelastic behavior of soft tissues. These include Kelvin-solid models, Schapery's Theory, and the Modified Superposition method [38]–[42]. Among different types of Kelvin-solid models, the standard linear solid (SLS) and Prony Series models have given the best predictions of viscoelastic responses under quasi-static conditions [39], [43]. However, these models have never been used to predict the load-relaxation response of the whole trunk. As such, the second purpose of the current study was to evaluate different viscoelastic modeling approaches for characterizing these responses. We hypothesized that available viscoelastic models would have differing success in characterizing these responses, with better predictions from Kelvin-solid models.

## Materials and Methods

### Ethics Statement

Prior to any data collection, all participants provided informed consent by reviewing and signing a consent form that described the aims and procedures of the study. The study procedures, including the consent form, were approved by the Virginia Tech Institutional Review Board.

Ten healthy young adults with no self-reported history of low-back pain participated after completing informed consent procedures approved by the Virginia Tech Institutional Review Board. Participants included five males with mean (SD) age, stature, and body mass of 24.4 (4.2) yr, 179.9 (6.9) cm, and 71. (7.3) kg, respectively; corresponding values for the five females were 23.8 (2.6) yr, 164.4 (3.9) cm, and 57.9 (5.1) kg. A relatively young set of participants (from 18–29 yr) was included to avoid potential influences related to age.

Each participant completed five experimental sessions, one for each of five levels of lumbar flexion including 30, 40, 60, 80, and 100% of the flexion-relaxation (FR) angle (see below). These flexion levels were used to cover a wide range of potential exposures, and the lower level was increased to 30% of FR angle based on pilot results that indicated exposure to 20% of FR angle was insufficient to capture viscoelastic properties. At least three days separated consecutive sessions, and the presentation order was counterbalanced using 5×5 Latin Squares (one for each gender). Sessions were conducted before 9:00 am to minimize effects of cumulative daily loading.

Lumbar flexion angle was measured using inertial measurement units (IMUs: Xsens Technologies XM-B-XB3, Enschede, Netherlands). IMUs were placed on the skin using medical-grade, double-sided tape, over the spinous processes of T12 and S1, and sampled at 100 Hz. Electromyography (EMG) of the Longissimus and Rectus Abdominus muscles was collected using bipolar Ag/AgCl surface electrodes and previously reported electrode placements [2], [44]. Specifically, electrodes were placed over the muscle belly at the L3 level and ∼3 cm lateral to midline at the level of the umbilicus, to measure activity of Longissimus and Rectus Abdominus muscles, respectively. Raw EMG data were preamplified (x100) near the collection site, and signals were then bandpass filtered (10–500 Hz) and amplified in hardware (Measurement System Inc., Ann Arbor, MI, USA) before being sampled at 1000 Hz.

After instrumentation, each participant stood in a rigid metal frame and straps were used to restrain the pelvis and lower limbs. In a preliminary session for each participant, FR angle was measured using procedures similar to an earlier study [2]. Briefly, participants flexed their trunk slowly to full passive trunk flexion (∼5 sec) and slowly returned to the upright standing posture (∼5 sec). FR angle was defined as the lumbar flexion angle, near the end of the range-of-motion, with minimal EMG. FR angle measurements were done three times, and the largest FR angle from the three trials was used as the reference for specifying flexion exposures in the experimental sessions. To minimize within-subject variability in FR angles due to creep-dependent changes [4], FR angles were desired at a relatively fixed level of creep deformation. This was achieved by inducing near-maximal (asymptotic) creep deformation of the trunk prior to obtaining FR angles. Specifically, participants adopted full passive trunk flexion for four minutes, which was expected to induce >90% of maximal creep [31]. All participants successfully developed passive tissues creep during the 4-min flexion exposures, and which ranged from 2.0 to 8.2 deg. Mean (SD) FR angles (measured after creep exposures) were 58.2 (12.0) deg across all participants. Of note, these creep exposures and FR angle measurements were performed only during the preliminary sessions.

**Figure 1:**
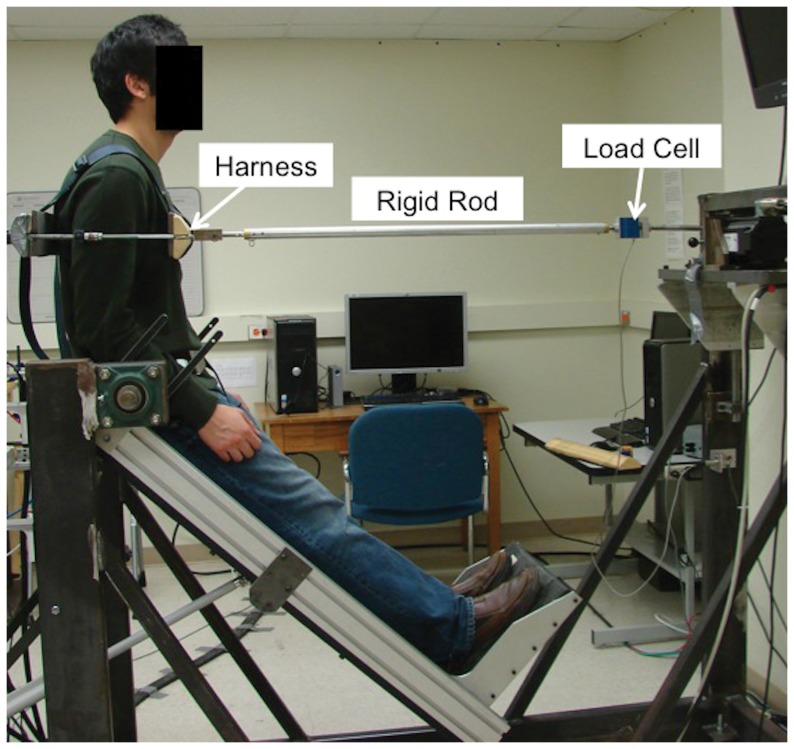
Experimental setup for load-relaxation test (60% FR angle condition illustrated).

While standing in the rigid frame with their pelvis restrained, trunk flexion was induced by rotating the pelvis and lower extremities, as a group, forward/upward, with an angular velocity = ∼3 deg/sec (Figure 1). Thereby, passive lumbar tissues were stretched and an external extension moment was produced. A footrest with adjustable height was used under the feet to position the L5/S1 joint at the frame's rotational axis. Participants' trunks were constrained at the T8 level using a rigid harness-rod assembly, which ensured that the trunk was maintained roughly upright. While the lower extremities were raised (loading phase), during the flexion exposure (load-relaxation phase), and while the lower extremities were lowered (unloading phase), forces due to passive tissues stretching were measured continuously (1000 Hz) using a load cell (Interface SM2000, Scottsdale, AZ, USA) on the harness-rod assembly. All data obtained (load cell, EMG, and IMUs) were collected synchronously using a LabVIEW™ virtual instrument (National Instruments, Austin, TX). EMG measures (as described above) were used as biofeedback to minimize voluntary muscle activation throughout these procedures, thus ensuring that measures were predominantly reflecting passive tissue properties. Participants also maintained a consistent head posture (facing forward and looking at a monitor). Flexion exposures lasted 16 minutes, which was considered sufficient to capture the majority of load-relaxation [36] and also be well tolerated by participants. For all calculations (see below), lumbar flexion angle measured from IMUs was used. Since the lower extremities and pelvis were restrained to the frame (and thus rotated together), and the upper torso remained upright, only lumbar motion segments were expected to be free to rotate in the sagittal plane. To confirm that target lumbar flexion angles were achieved, and that substantial contributions from thoracic or pelvic rotations were not present, lumbar flexion angles (measured directly from the IMUs) were compared with the angles over which the legs were raised (measured from a scale on the frame). Across conditions these angles differed by <5%.

**Figure 2:**
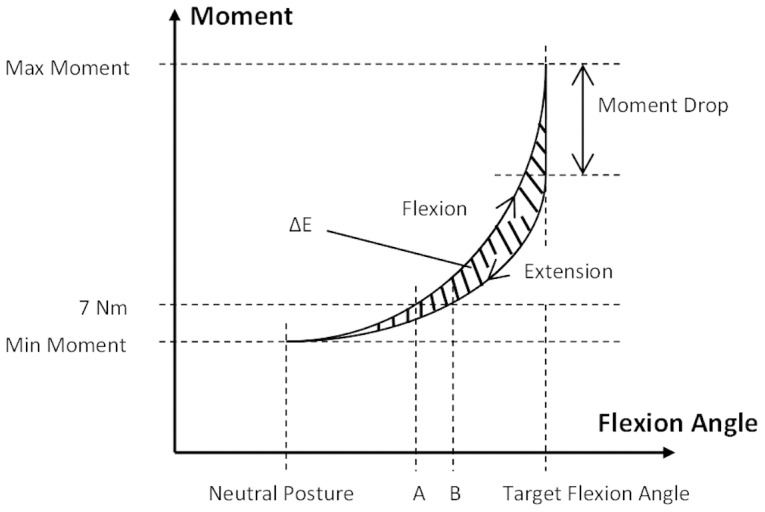
Illustration of a hysteresis loop. The highlighted area (ΔE) denotes the dissipated energy; NZ in flexion (extension) is the distance between point A (point B) and the neutral posture. Target lumbar flexion angle = 30, 40, 60, 80, or 100% FR.

Several direct or derived outcome measures were obtained for the trunk: 1) initial moment, 2) moment drop, 3) neutral zone (NZ), 4) viscoelastic state, and 5) viscoelastic model parameters characterizing the viscoelastic (load-relaxation) behaviors. Initially, the exposure periods were divided into the three phases noted above (loading, load-relaxation, and unloading). Trunk moments were determined from the measured force (load cell) and associated moment arm (measured vertical distance between the rod and L5/S1 center of rotation). Three-second windows at the start and end of the load-relaxation phase were used to calculate the initial moment and moment drop. Loading (flexion) and unloading (extension) phases were used to estimate the NZ (Figure 2), a region over which little resistance exists against external forces or moments [45]. The NZ was defined specifically as the portion of the lumbar range of motion around the neutral (upright) posture where the slope of the lumbar flexion angle-moment curve was <0.1 Nm/deg and the passive moment was<7 Nm [46]. For each participant, the NZ range was divided by the FR angle to yield a normalized NZ for each flexion exposure, and the percentage change from the pre-exposure value was obtained.

Total energies for flexion (*E*_1_) and extension (*E*_2_) were calculated from areas under the flexion-angle-moment curves in the loading and unloading phases, respectively, and these were used to determine dissipated energy: Δ*E* = *E*_1_–*E*_2_ (Figure 2). Subsequently, the ratio of hysteresis/energy input (RE), which describes the viscoelastic state [47], was estimated as Δ*E*/*E*_1_. For a pure elastic material, RE = 0, and for a pure viscous material RE = 1 [47], [48].

To characterize trunk viscoelastic behaviors, four common types of viscoelastic models of varying complexity were used, with the load-relaxation equations for each provided below:

### SLS model (Figure 3)



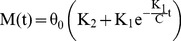
(1)where 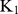 and 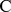 are, respectively, stiffness and damping of torsional spring and damper components in series (Maxwell component), and 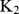 is the stiffness of a parallel torsional spring [49]. 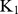 and 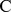
represent viscous responses to deformation, and 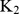 is the steady-state stiffness once the material is totally relaxed. 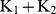 is the instantaneous stiffness, and the relaxation time constant (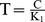) shows the rate of moment relaxation.

**Figure 3:**
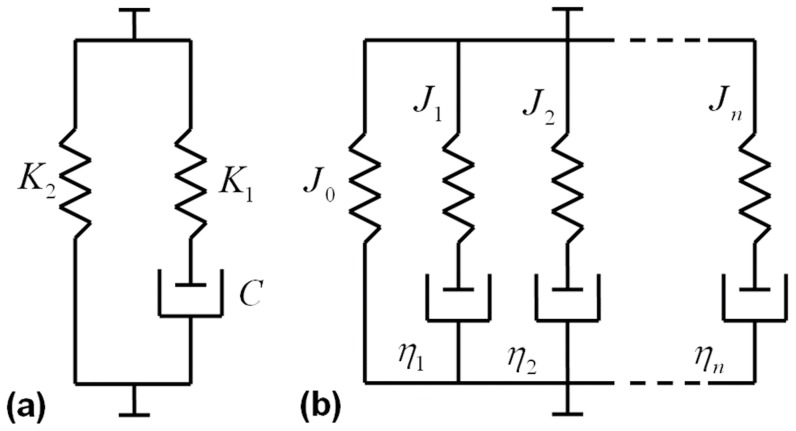
Illustration of two Kelvin-solid models: (a) SLS model (b) Prony Series model. Each spring and damper in series represents a Maxwell model. For clarity, linear rather than rotational components are illustrated.

### Prony Series (Figure 3)



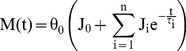
(2)where 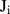, and 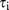 (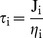) are respective stiffness and relaxation time constants from each spring and damper in the *i* ^th^ Maxwell component of the Wiechert model. 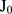 is the is the steady-state stiffness once the material is totally relaxed, and 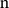 is the number of Maxwell components in the model [39]. Here, values of 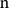 = 2, 3, and 4 were considered.

### Schapery's Theory



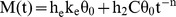
(3)where 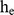
and 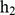 are angle-dependent constants, 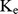 is the torsional stiffness at equilibrium (final data point), and 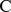 and 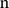 are constants that were derived by curve fitting [38], [50].

### Modified Superposition Method



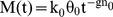
(4)where 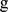 is an angle-dependent constant, 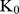
is the torsional stiffness at the beginning of load-relaxation, and 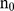 is the initial relaxation rate obtained by curve fitting [38], [42].

These equations (models) were derived assuming a constant lumbar flexion angle = 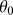 and using established procedures [38], [49], [51]. Model parameters were estimated for each exposure (i.e., each participant in each lumbar flexion angle) by minimizing least-squared errors in predicted moments within the load-relaxation phase. Subsequently, model prediction quality was evaluated using the mean, across participants, of coefficients of determination (*R* ^2^) and root-mean-square errors (RMSE) obtained for each exposure. Model prediction quality using the Prony Series model was comparable using 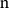 = 2, 3, and 4, and thus the simplest equation (i.e., 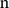 = 2) was used in the remainder of this work.

Separate mixed-factor, repeated-measures analyses of variance (ANOVAs) were performed to evaluate the effects of lumbar flexion angle and gender on each of the direct and derived measures. Only the relevant SLS model parameters (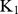,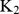, 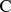, 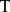, and 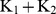) were analyzed in this way, to assess potential nonlinearity in elastic and viscous properties, since their interpretation is relatively straightforward versus parameters within the other models. Post-hoc comparisons between flexion exposure levels were done, where relevant, using Tukey's HSD. Effects of lumbar flexion angle on direct outcome measures (i.e., initial moment, moment drop and changes in NZ) were also explored using linear and nonlinear curve fits to mean values, and these were evaluated based on coefficients of determination (*R* ^2^). As several such curves should logically include the origin (e.g., zero flexion yields zero moment), the origin was included as an additional data point. However, SLS model parameter values near 0% FR were not extrapolated, since in this region (i.e., the NZ) rotational stiffness is substantially smaller than elsewhere [46], [52]. Statistical significance was concluded when *p*<0.05, all analyses were performed using JMP (Version 9, SAS Institute Inc., Cary, NC, USA), and all summary statistics are given as means (SD). Incomplete data were available for four trials involving 30% FR exposures, during which clear moment changes over time were not evident, and results from one 100% FR trial were excluded as clear outliers (studentized residuals).

A sensitivity analysis was performed to assess the effects of each model parameter with respect to describing viscoelastic behavior of the trunk. Sensitivity coefficients were calculated as [53]:
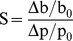
(5)where 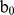 is the nominal value (mean value across all trails) of a relevant outcome measure (i.e., moment drop and initial moment), and 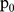 is a given model parameter; 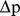 is the range of the model parameter across all trials; and, 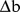 is the range in the predicted outcome measure (i.e., change in moment drop or initial moment prediction) that results from changing the given model parameter over 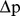 while all other model parameters are kept at their nominal values. All model-based calculations were performed in MATLAB™ (MathWorks, Natick, MA, USA).

## Results

**Figure 4:**
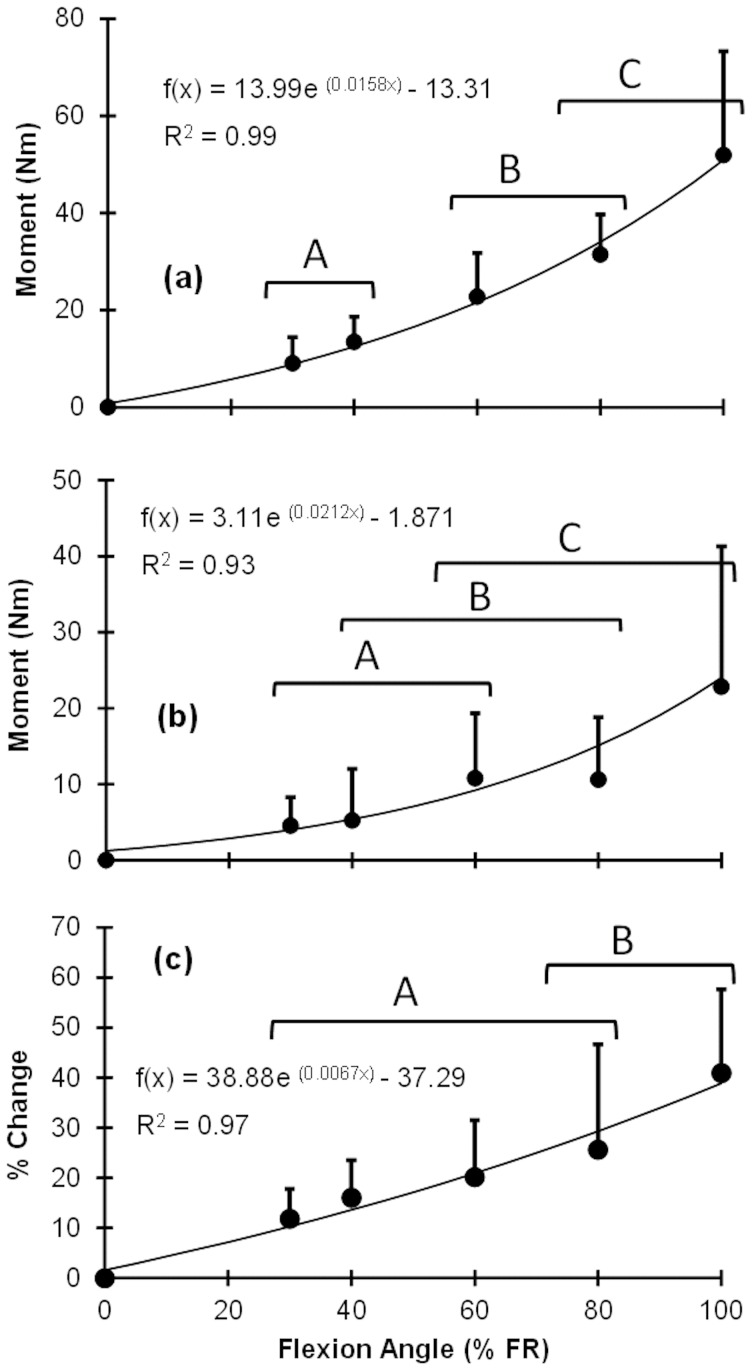
Effects of lumbar flexion angles on direct outcome measures: (a) initial moment, (b) moment drop, and (c) percentage change in normalized NZ. Post-hoc groupings are indicated by brackets and letters, and best-fit exponential relationships are provided.

There were significant effects of lumbar flexion angle on initial moment (*F*_(4,25)_ = 29.51, *P*<0.0001), moment drop (*F*_(4,23)_ = 9.08, *P*<0.0001), and changes in normalized NZ (*F*_(4,21)_ = 5.82, *P*<0.0025). All three measures increased with lumbar flexion angle (Figure 4), and each of the relationships with lumbar flexion angle was well characterized by exponential functions (*R* ^2^>0.93). Viscoelastic state (RE) overall was 0.42 (0.15), indicating a mix of elastic and viscous behaviors, and was not affected by lumbar flexion angle (*F*_(4,22)_ = 0.39 *P* = 0.81). Gender had no main or interactive effects on any of these outcome measures (*P*>0.11).

**Figure 5:**
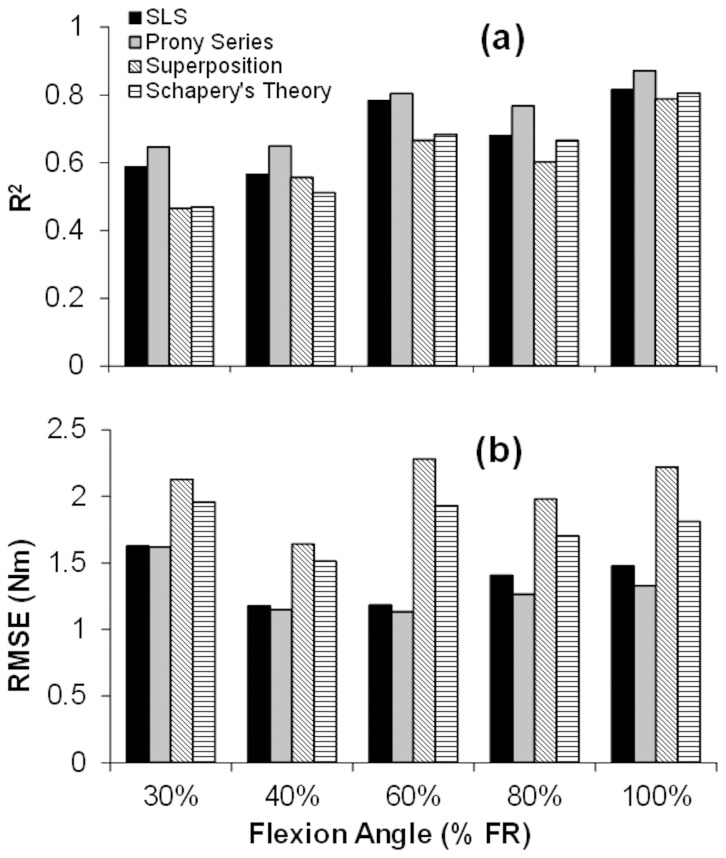
Mean measures of viscoelastic model prediction quality: (a) *R* ^2^ and (b): root-mean-square errors (RMSE).

The different models exhibited different levels of prediction quality (as based on *R* ^2^ and RMSE) and some levels of dependency on lumbar flexion angle (Figure 5). Overall differences in *R* ^2^ and RMSE between the SLS and Prony Series models were negligible (8 and 5%, respectively), and these two exponential models produced better predictions than the two power models (i.e., Schapery's Theory and the Modified Superposition Method). While RMS errors were consistent across lumbar flexion angles, *R* ^2^ generally increased with angle for each model.

**Figure 6:**
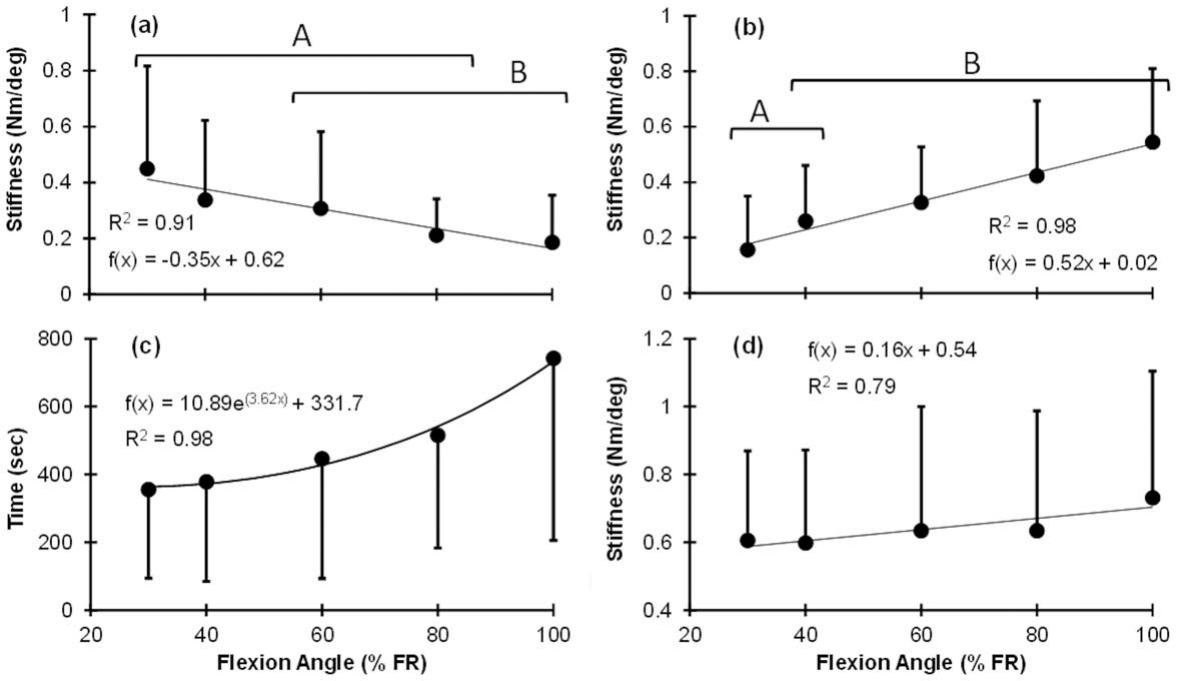
Effects of lumbar flexion angle on SLS model parameters: (a): stiffness of Maxwell component = K_1_, (b): parallel stiffness = K_2_, (c): relaxation time constant = T, and (d): instantaneous stiffness = K_1_+K_2_. Post-hoc groupings are indicated by brackets and letters, and best-fit relationships (linear or exponential) are provided.

Lumbar flexion angle significantly affected the 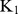
(*F*_(4,24)_ = 3.84,*P* = 0.0154), and 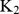
(*F*_(4,23)_ = 6.96, *P* = 0.0008) parameters within the SLS model; 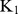 decreased and 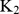 increased with lumbar flexion angle (Figure 6). 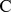, 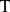, and 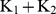, in contrast, were not affected by lumbar flexion angle (*P*>0.10), and gender had no main or interactive effects on any of the SLS model parameters (*P*>0.07). 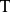 and 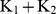 tended to increase with lumbar flexion angle, while 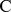 remained quite consistent across all lumbar flexion angles with mean (SD) = 111 (107) Nms/deg. Parameters obtained for the other models at specified lumbar flexion angles are presented in Table 1.

**Table 1:** Mean (SD) values of estimated parameters for different viscoelastic models with respect to lumbar flexion angle (SLS model parameters are shown in Figure 6).

Model parameters (Units)	Lumbar flexion angle (percentage of FR angle)				
	30%	40%	60%	80%	100%
**Prony Series**					
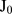 (Nm/deg)	0.07 (0.14)	0.19 (0.12)	0.19 (0.23)	0.24 (0.28)	0.23 (0.25)
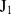 (Nm/deg)	0.40 (0.36)	0.25 (0.30)	0.49 (0.46)	0.36 (0.38)	0.36 (0.24)
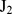 (Nm/deg)	0.10 (0.19)	0.17 (0.28)	0.21 (0.17)	0.21 (0.26)	0.34 (0.38)
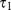 (sec)	12.6 (15.4)	20.6 (17.3)	7.6 (12.1)	9.8 (10.3)	8.8 (11.1)
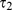 (sec)	1058.4 (1352.5)	1030.6 (704.7)	764.4 (632.5)	1690.6 (1323.1)	1704.5 (1491.3)
**Schapery's Theory**					
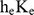 (Nm/deg)	0.15 (0.01)	0.15 (0.01)	0.26 (0.01)	0.23 (0.01)	0.54 (0.01)
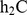 (Nm/deg*sec)	0.62 (0.54)	0.62 (0.54)	0.74 (0.58)	0.60 (0.34)	0.68 (0.42)
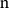 (dimensionless)	0.28 (0.40)	0.42 (0.57)	0.32 (0.23)	0.24 (0.25)	0.23 (0.16)
**Modified Superposition**					
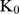 (Nm/deg*sec)	0.522 (0.272)	0.524 (0.265)	0.560 (0.232)	0.564 (0.227)	0.749 (0.386)
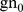 (dimensionless)	0.29 (0.39)	0.09 (0.12)	0.09 (0.07)	0.07 (0.06)	0.06 (0.03)

From the sensitivity analyses, several dependencies were evident (Table 2). Some parameters (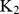, 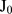, and 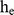) were purely related to elastic behavior (initial moment), while others (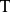, 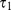, 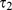, 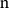, and 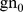) were purely related to viscous responses (moment drop) of soft tissues. The remaining parameters (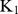,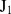, 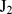, 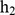, and 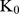) were related to both elastic and viscous behaviors. Of note, the moment drop sensitivity coefficient of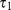 (i.e., the smaller relaxation time constant in the Prony Series model) was several orders of magnitude smaller than that of 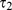 (i.e., the larger relaxation time constant); hence, the larger relaxation time constant describes more of the moment drop.

**Table 2:** Dimensionless sensitivity coefficients for the four models with respect to initial moment and moment drop.

Model parameters	Sensitivity coefficient	
	Initial moment	Moment drop
**SLS model**		
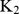	0.66	0.00
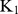	0.31	0.67
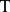	0.00	0.14
**Prony Series**		
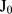	0.27	0.00
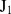	0.52	1.27
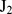	0.29	0.38
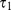	0.00	2.5 e-5
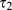	0.00	0.09
**Schapery's Theory**		
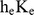	0.36	0.00
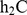	0.95	2.03
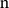	0.00	0.03
**Modified Superposition**		
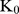	0.84	1.15
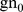	0.00	0.24

## Discussion

Nonlinearity in both elastic and viscous properties of the trunk was clearly evident. This was apparent both from nonlinear changes in initial moment and moment drop with lumbar flexion angle and the angle-dependency of SLS model parameters (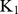
and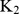). Here, exponential increase in moment drop and 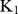 reduction with lumbar flexion angle demonstrated nonlinearity in the viscous behavior of the trunk. Moreover, relaxation rate (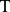
in Equation 3) increased with lumbar flexion angle (Figure 6). Although this change was not significant, it suggests that more time is required for the initial moment to relax at larger lumbar flexion angles. While there is previous evidence of nonlinear viscoelastic behaviors of spinal soft tissues [33], [37], the current work presents new evidence for nonlinearity in the whole trunk. The flexion distribution among thorax and lumbar components was not controlled here. However, and as suggested by previous work [54], [55], and our direct measurement of lumbar angle most of the flexion likely occurred in the lumbar spine.

Estimated elastic and viscous properties here are comparable with previous reports. For elastic behavior, the magnitudes of initial moment versus lumbar flexion angle (i.e., instantaneous moment-angle relationship) were similar to those in previous studies [56], [57]. Changes in the initial moment (and 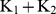) with lumbar flexion angle also showed the same nonlinear moment-angle relationship that has been found earlier [58], [59]. For viscous behavior, the mean (SD) value of moment drop during load-relaxation periods was 41 (22)% across all five exposure conditions. Earlier *in vitro* studies reported a ∼48% reduction in flexion reactive moment of lumbar spine motion segments [16] and ∼27% reduction in passive muscle force [19], [30], [60] after 16 minutes of loading. (Approximate values were derived using interpolation.) Moreover, the current mean (SD) value of the required time for a 90% drop relative to the initial moment was 5.9 (3.7) minutes, similar to values of ∼5 minutes for spinal motion segments and ∼9 minutes for passive muscles [60].

According to observed values of moment drop and relaxation duration, it is possible to infer which tissue components of the trunk are predominant in providing viscous behavior. We consider two parallel systems to be responsible for generating the reactive moment: 1) spinal motion segments (i.e., vertebrae, disc, facets and ligaments), and 2) passive tissues integrated within muscle units (i.e., tendon, epimysium, p erimysium, and endomysium). Optimal lengths of the active force-length relationship of trunk-extensor muscles occur at lumbar angles close to full flexion [61]–[63], and passive tension developed in muscles typically starts at/near this optimal length and increases as length increases. However, other studies have indicated smaller lumbar flexion angles corresponding to the peak trunk extension moment [64], [65], and this discrepancy may be related to differences in experimental methods used and between individuals (i.e., different ages or genders). Thus, at less extreme lumbar flexion angles (30–100% of FR angle) the contribution of passive muscle forces was assumed to be relatively small. In this study, mean maximum flexion exposure during load-relaxation (100% of FR) was equal to 87% of the mean full lumbar flexion angle. Accordingly, it was expected that spinal motion segments (rather than passive muscle stiffness) were predominant in providing the measured reactive moment. This was also supported by the fact that measured initial moments here are comparable with previously reported values for isolated spinal motion segments (without muscles) [16], [66]. As such, for angles smaller than FR, a majority of the moment drop should thus result from viscoelastic behavior of spinal motion segments. Of note, this reduction in stiffness should be compensated by additional muscle activities, such as when performing a task following a prolonged period of flexion. Extrapolating from the current research and previous modeling results [7], this extra muscle activity could substantially increase the internal load on the spine, up to ∼600 N in extreme cases. Results here, though, were not sufficient to explain in detail the passive moment allocation among different components of spinal motion segments. For instance, ligaments might contribute to passive moment in trunk flexion exposures, and consequently to the passive moment drop during the load-relaxation period. As such, a reduction in ligament forces can reduce the imposed forces on spinal motion segments. However, it was beyond the scope of the current study to explore the force/moment distribution among different passive components within spinal motion segments.

When the trunk is flexed, passive tissues resist the external moment, yet this resistance is small for deformations near the NZ [52]. Panjabi [67] suggested that an increase in the NZ reflects instability and an increased LBD risk, and it may also be a sensitive parameter for defining the onset of spinal injuries [68]. According to Yamamoto [69], the NZ for flexion is 8.8 degrees for the L1-S1 spine, which is comparable to the current mean (SD) of 10.5 (5.5) degree here prior to flexion exposure. Rotational displacements of other spinal motion segments superior to the lumbar vertebrae likely account for the difference between the NZ measures in the current *in vivo* study and previous *in vitro* studies. In agreement with the effect of lumbar flexion angle on viscoelastic behavior, pre- and post-exposure NZ differences increased exponentially with lumbar flexion angle. Previous *in vivo* studies have reported an increase in spinal motion segment laxity after prolonged and cyclic flexion [70], [71]. These studies measured the neuromuscular neutral zone (NNZ), which is the amount of rotational displacement applied to the lumbar spine before muscle activity increases the stiffness of the intervertebral joints. Though NNZ and NZ might be different in magnitude [71], it is expected that they are closely related to each other, and results from the current study confirmed that flexion exposures increase the NZ as well. However, the present results regarding a nonlinear increase in NZ changes with lumbar flexion angle have not, to our knowledge, been previously quantified. An increase in NZ following prolonged flexion exposure suggests that the LBD risk may increase as well, and that the increase in LBD risk depends on the extent of lumbar flexion angle involved.

Comparing moment-angle curves before and after flexion exposures demonstrated that trunk soft tissues generated lower reactive moments for an identical lumbar flexion angle after exposures. This phenomenon of a hysteresis loop during loading and unloading has been shown in previous *in vitro* studies on soft tissues. In these, RE values have been reported equal to ∼0.2 for intervertebral discs under axial compression [72], and between 0.1 and 0.59 for spinal ligaments in load-relaxation [48]. However, no evidence could be found regarding RE for flexion exposure of the whole trunk, especially at different lumbar flexion angles. Here, an almost constant RE value of 0.42 (0.15) was found at different lumbar flexion angles, with no clear increasing or decreasing trend, suggesting an identical viscoelastic state for the whole trunk over a wide range of lumbar flexion angles. Because both elastic and viscous properties change with lumbar flexion angle, these RE outcomes do not contradict our earlier results regarding nonlinearity in viscoelastic properties. Rather, the RE results suggest that elastic and viscous properties change in parallel and such that the overall viscoelastic state of the trunk is independent of lumbar flexion angle.

Assessing differences related to gender was not a main focus of this study, and which was likely underpowered in this respect. Indeed, no significant differences were evident, though some suggestive results were found. Overall, males exhibited greater flexion stiffness, with 15% higher initial moments, 6% lower maximum lumbar flexion angles, and 7% lower FR angles. The same qualitative difference in stiffness between genders was observed from the SLS model, where 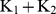 was 6% greater among males. In partial agreement with our findings, greater flexibility in females has been previously reported for trunk flexion [73]–[75].

**Figure 7:**
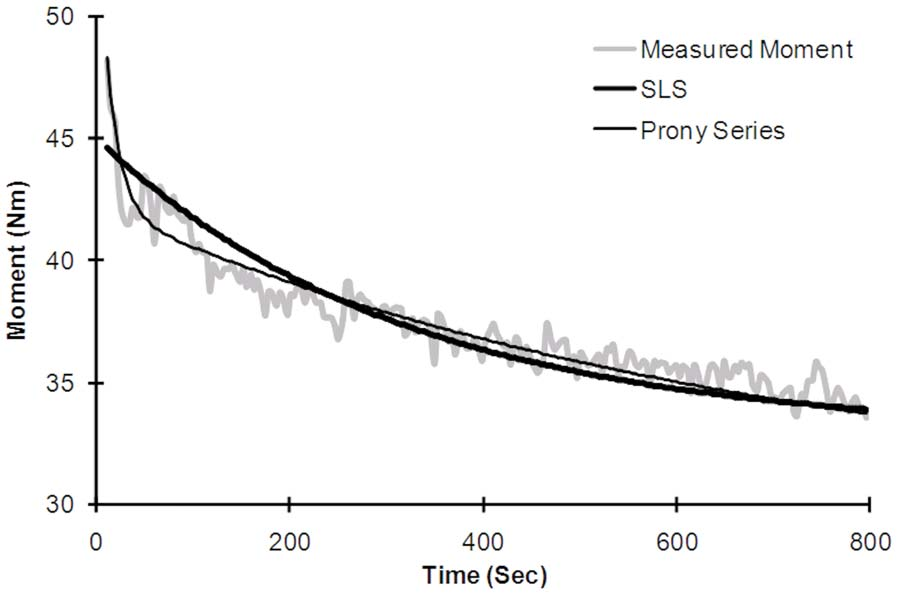
Fast and slow phases of moment drop during the load-relaxation period. Representative data are shown, and which indicate the advantage of the Prony Series over the SLS model for predicting measured behaviors. Results are for a 100% FR exposure.

We evaluated different viscoelastic modeling approaches in terms of their ability to characterize the load-relaxation responses of the human trunk. Both the Prony Series and SLS models, using exponential equations, were more effective for describing viscoelastic behavior of the trunk than the two power models. Predictions from these two exponential models, however, differed slightly in how they described the immediate moment drop (i.e., at the beginning of the load-relaxation period). From inspection of load-relaxation graphs, distinct fast and slow phases can be identified in most, with the transition occurring in roughly the first 30-60 seconds of exposure (representative data shown in Figure 7). These two phases are more easily distinguishable when exposure was to larger lumbar flexion angles. Similar dual-phase results have been reported for the creep behavior of spinal motion segments [41], with two specific creep rates: fast-rate creep, immediately after loading (from 0 to 1 minute of exposure); and slow-rate creep for the remaining exposure duration (from 1 to 480 minutes of exposure). Hence, the Prony Series model, with two relaxation time constants (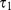
and 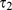),may be more appropriate than the SLS model for predicting load-relaxation behavior, especially in response to larger lumbar flexion angles (see also Figure 5, which showed larger RMSE differences between the two models with increasing lumbar flexion angle). Results from the sensitivity analysis confirmed the benefits of adding an additional, shorter relaxation time constant (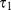) in the Prony Series model, though the sensitivity coefficient of 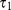
was quite small.

An important potential limitation of the current study is related to the (in) accuracy in measuring *in vivo* viscoelastic properties. It is challenging to measure viscoelastic properties *in vivo*, with two of the more substantial problems related to the relatively modest changes in moment during load-relaxation and the unavoidable presence of uncontrolled body movements. The former was particularly problematic for small exposure angles, and as noted earlier four trials involving 30% FR exposures were discarded due to insufficiency in capturing viscoelastic properties. These effects account, at least in part, for the larger variability within each exposure (larger RMSE) compared to *in vitro* studies. To minimize the latter source of error, voluntary movements were controlled (to the extent feasible) during data collection, both visually and using EMG. Additional analysis of the EMG data was done, and mean values of raw EMG data were not significantly different between the first and the last minute of load-relaxation (from paired *t*-tests, *P* = 0.45 and *P* = 0.25 for the extensors and flexors, respectively). Both EMG values, though, decreased slightly (less than ∼5%) over the exposure period, perhaps due to a decrease in co-contraction with prolonged exposure. This decrease in muscle activity, in any case, likely led to some overestimation of moment drop and underestimation of viscous stiffness (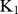). Further, the gluteal muscles have a primary role in hip and trunk extension, and an important effect in spine stability during gait [76]. The activity of these muscles, however, was not monitored during the present study due to limitations in placing the electrodes.

In summary, the current work can facilitate a better understanding of how the load distribution among passive and active trunk components changes during prolonged flexion exposures. The current experimental setup isolated the effects of lumbar flexion angle independent of variation in gravitational loads and trunk muscle activity; specified lumbar flexion angles were achieved by raising participants' legs, rather than by having participants maintain forward flexion of the trunk. Any variability or potential confounding induced by muscle activity, inaccurate posture maintenance, or fatigue was thereby minimized. The results described an angle-dependent and nonlinear relaxation behavior of the human trunk. Measured load-relaxation more likely arose from viscoelastic behavior of spinal motion segments, rather than passive muscles. Furthermore, viscoelastic responses were characterized using different types of models and material properties were derived, for which Kelvin-solid models more efficiently described load-relaxation behavior than other models. Such viscoelastic material properties can be used to predict trunk behaviors and lumbar mechanics in response to prolonged flexion exposures, for example by incorporation within larger-scale biomechanical models. In the occupational domain, diverse tasks involve prolonged exposure to flexed postures; as such, the current results may help in future efforts to control work-related LBDs.
